# Cytogenomics of Feline Cancers: Advances and Opportunities

**DOI:** 10.3390/vetsci2030246

**Published:** 2015-08-31

**Authors:** Rachael Thomas

**Affiliations:** Department of Molecular Biomedical Sciences, College of Veterinary Medicine & Center for Comparative Medicine and Translational Research, North Carolina State University, Raleigh NC 27607, USA; E-Mail: rachael_thomas@ncsu.edu; Tel.: +1-919-513-0922; Fax: +1-919-513-7301

**Keywords:** cat, feline, genomics, chromosome, cytogenomics, cancer, comparative, fibrosarcoma, lymphoma, mammary carcinoma

## Abstract

Relative to the dog, integration of the cat into the “One Health” concept has been more restricted, particularly in the field of molecular oncology. Beyond the continual need to enhance the sophistication of feline healthcare *per se*, the unique spectrum of naturally-occurring cancers in the cat offers tremendous opportunities for comparative and translational advances that may have mutual benefit for human and veterinary medicine. The study of feline cancers additionally may generate new insight into underexplored aspects of tumor biology that are less accessible in other species, such as the relationship between chronic inflammation and neoplasia, and the role of viruses in malignant transformation. Several factors that have hindered molecular studies of feline cancers have now been surmounted, with the most fundamental step forward coming from the development of a high-quality reference genome sequence assembly for the cat. This article reviews landmark studies that have led to our current appreciation of feline genome architecture, and outlines techniques used in cancer cytogenomics, from conventional karyotyping analysis through to the development of genomic microarrays and beyond. A summary of progress in the identification and characterization of chromosomal aberrations in feline cancers is provided using examples from studies of injection-site sarcomas, lymphomas and mammary tumors.

## 1. Introduction

The domestic cat offers a diverse range of opportunities to contribute to the “One Health” concept, which capitalizes on the integration of complementary human and veterinary biomedical research efforts for synergistic gain. Cats continue to grow in popularity as family-owned pets and benefit from increasingly comprehensive medical surveillance [1]. It has been estimated that one in three U.S. households owns at least one cat, equating to a total U.S. cat population of approximately 74 million and a total annual expenditure of approximately $7.4 billion on veterinary services [2]. Cats exhibit approximately 200 genetic diseases that are considered analogous to human disorders, many of which are rarely encountered in any other widely studied species [3]. The cat has served as a particularly powerful model system for studying facets of neuroscience, reproduction and both heritable and infectious disease, and the field of feline oncology offers several novel angles to the comparative medicine arena. The ability to explore these attributes from a molecular standpoint has become possible only relatively recently through intensive efforts to characterize domestic cat genome organization in detail. The combination of data from complementary approaches now allows us to explore the cat genome directly in context with that of humans and other key players in comparative biomedical research. In turn we may now begin to investigate in earnest the causes and consequences of genomic abnormalities associated with heritable and spontaneous disease in our feline companions.

## 2. Gross Karyotypic Organization in the Domestic Cat

The genome of the domestic cat (*Felis catus*, FCA) is organized into 18 pairs of autosomes and two sex chromosomes. Unlike in other mammals, where autosomes are numbered consecutively in descending order of size, standardized nomenclature in the cat takes into account both chromosome size and morphology. The karyotype comprises six autosomal subgroups termed A-F, with between two and four members in each. All but two autosomal pairs, cat chromosomes (FCA) F1 and F2, are biarmed, as are both sex chromosomes. There is substantial variation in chromosome size throughout the karyotype, from the largest (FCA A1, 240Mb) to the smallest (FCA E3, 43Mb) [4,5]. This marked diversity in size and morphology, broadly comparable to that of the human karyotype, means that chromosome identification in the cat is generally less challenging than in other key model species that exhibit more limited karyotypic variation. Gross similarity in the architecture of the human and domestic cat karyotypes has long been evident through direct comparison of their chromosome banding patterns [6,7]. These observations were reinforced by a series of molecular cytogenetic studies that indicated a remarkably limited degree of divergent evolution between the human and domestic cat genomes over the 92 million years since they shared a common ancestor, compared to the more extensive reshuffling evident in other model species [4,8,9,10,11]. The passage towards development of whole genome sequence assemblies for diverse mammals has since provided a far more comprehensive appreciation of conserved genome organization and potential mechanisms for karyotypic evolution. 

## 3. Generation of a Reference Cat Genome Sequence Assembly

The potential role of the domestic cat in comparative biomedical research was boosted substantially by the generation of a light (~two-fold) coverage feline genome sequence assembly of “Cinnamon”, a female Abyssinian, which captured ~65% of euchromatic sequence [12]. This formed part of a broader initiative funded by the National Human Genome Research Institute (NHGRI) to aid interpretation of the human genome sequence assembly, and to investigate patterns of sequence conservation, divergence and rearrangement between rodents, primates and other carnivores [13]. These studies showed that genome evolution in canine, ursine and murine lineages was driven strongly by interchromosomal translocation events, while intrachromosomal inversions were predominant in felids and also most primates [10,12]. A ~14x coverage whole genome assembly has recently become available for “Cinnamon”, spanning 2.35 Gb, with sequence annotation for almost 22,000 putative protein-coding cat genes [4,5]. This resource affords a fascinating insight into the genomic factors that provide the unique phenotypic attributes of the domestic cat, and firmly equips the cat to contribute to, and benefit from, a “One Health” approach to biomedical research. Among the most rapid and exciting advances in feline molecular medicine are now being made in the area of oncology.

## 4. Fundamentals of Cancer Cytogenomics

It is well established that the onset of a malignant phenotype is associated with the accumulation of both random and non-random genomic abnormalities [14,15]. Molecular characterization of numerous diverse human cancers has identified vast numbers of recurrent somatic abnormalities, of which a proportion have been shown to correlate with discrete clinicopathologic criteria [16]. The ability to screen individual patients for these genomic markers has revolutionized diagnostic and prognostic capabilities, refining historically broad tumor classifications into clinically predictive subcategories and paving the way towards personalized disease management strategies. “Genomic profiling” also provides insight into the molecular pathogenesis of disease, identifying gene dosage imbalances associated with functional disruption of regulatory pathways, revealing key mechanistic processes underlying neoplastic transformation and progression and highlighting opportunities for targeted therapeutics.

Techniques for molecular cytogenomic evaluation of tumors fall broadly into two categories (see [17] for a detailed review). Direct techniques, such as fluorescence *in situ* hybridization (FISH) analysis, enable both structural and numerical assessment of small numbers of discrete genomic loci (typically < five) within individual cells. In contrast, indirect methods, such as comparative genomic hybridization (CGH) analysis, permit semi-quantitative measurement of mean DNA copy number within a tumor cell population at many thousands of defined intervals selected throughout the genome, but cannot detect balanced structural rearrangements. Since cancers typically exhibit a combination of both structural and numerical aberrations coupled with cell-to-cell heterogeneity, these highly complementary approaches are often used in tandem. Modified protocols allow their application to challenging specimens, such as those where the availability of clinical material is limited, or where the integrity of the sample is compromised, such as archived histologic specimens. This opens up a multitude of possibilities for retrospective studies of case materials for which detailed clinical histories are available.

The concepts of cancer cytogenomics are well established in human medicine, and enormous strides have been made in translating the same fundamental principles to domestic dog cancers. This has revealed remarkable conservation of discrete molecular alterations in human and canine counterparts of the same cancer (reviewed in [18,19]), providing strong evidence in support of a shared pathogenesis. Progress in the feline field is now gaining momentum, particularly in those areas in which the cat offers a novel perspective that has not been explored in other model species.

## 5. Injection-Site Sarcoma

It is now 25 years since concerns were first raised over the increasing number of reports of malignant lesions arising at routine vaccination sites in cats [20]. The masses, predominantly fibrosarcomas, were observed most commonly in association with inflammatory reactions following the use of adjuvanted vaccines against rabies and feline leukemia virus. Vaccine-site sarcomas typically exhibit a rapid, aggressive clinical course and a bleak long-term prognosis. Treatment often involves radical surgical intervention due to the highly invasive nature of the disease, despite which localized post-surgical recurrence is common [21]. Since wide surgical margins markedly reduce the risk of tumor regrowth, there has been widespread encouragement for a transition away from traditional interscapular and femoral vaccination sites where adequate resection is highly challenging. The introduction of non-adjuvanted vaccines has also gone some way to limit the impact of vaccine-site sarcoma; however it remains a substantial clinical concern in feline medicine [22]. 

Interestingly, histopathologically similar tumors have also been described, infrequently, in dogs, ferrets and rabbits [23,24]. Furthermore, this phenomenon is not restricted to vaccination, since there are reports, albeit rare, of apparently related tumors manifesting in conjunction with injection of other fluids, in response to non-absorbable suture materials and even following microchipping procedures [21]. This has led to increased usage of the term “injection-site sarcoma” (ISS). Additionally, ISS shares many features with lesions occurring following ocular trauma in cats, and also with tumors arising in people in response to prolonged contact with foreign materials, such as artificial implants [22,25]. Taken in combination, these observations provide intriguing evidence for an association between persistent inflammation and malignant transformation. More than two decades after the first description of ISS, however, there remains no solid, detailed explanation for their underlying etiology, and they remain somewhat of an enigma [26,27]. 

Recognition of the etiology, incidence and impact of feline ISS has been challenged by difficulty in conclusive distinction between these sarcomas and those arising spontaneously without an apparent underlying injection-related origin. ISS typically are more locally invasive and prone to local recurrence than fibrosarcomas of spontaneous origin (non-ISS). Consequently, ISS generally warrant highly extensive surgical margins followed by regular and meticulous monitoring for recurrence, while non-ISS tumors may respond favorably to more conservative clinical management [27,28]. The ability to distinguish ISS from non-ISS at diagnosis therefore has far-reaching implications for patient, owner and clinician; however their extensive clinicopathologic similarity often confounds prompt and unequivocal identification. Distinction between these subtypes currently involves combinatorial consideration of multiple histopathologic features in context with clinical history and anatomical location, and often remains subjective and inconclusive. Currently there are no robust and broadly consistent diagnostic features for distinction between these subtypes. Advances in feline genomics now offer a potential molecular means to address this need and comprehend the factors that underlie the differences in their biological behavior.

Prior to the genomics era, conventional cytogenetic studies identified highly disrupted karyotypic organization in feline fibrosarcomas, with frequent hyperdiploidy and broad cell-to-cell heterogeneity [29,30,31,32]. A putative link has since been reported between amplification of feline satellite DNA sequences and the onset of genomic instability in fibrosarcomas, likely through disruption of normal centromere activity and the formation of aberrant marker chromosomes [33]. A subsequent study of five established ISS cell lines by classical enumeration of banded chromosome preparations also showed tremendously complex karyotypic reorganization, with a range of 19 to 155 chromosomes per cell, as compared to the normal 2n = 38 [34]. None of these studies, however, included detailed assessment of the composition of these aberrant chromosome structures.

Towards this goal, a microarray platform was developed to permit CGH-based identification of recurrent genomic DNA copy number aberrations (CNAs) in feline tumors [35]. This “aneuploidy array” comprised 210 large-insert clones representing loci anchored at ~15 Mb intervals throughout the initial 2x-coverage cat genome sequence assembly. Microarray-based profiling of 46 retrospective primary feline fibrosarcomas identified an extensive range of highly recurrent CNAs, consistent with the high level of genomic instability identified in earlier chromosome-based studies. Targeted FISH analysis of tumor chromosome preparations demonstrated broad heterogeneity in the patterns of structural and numerical aberrations evident both within and between cases. Remarkably, combined CGH and FISH analyses showed that a subset of genomic regions exhibited in excess of 30 copies within individual tumor cells, which were distributed across as many as ten different chromosome structures. Also of note, ISS cases showed non-random reorganization of FCA E1 in conjunction with copy number amplification of a discrete region on FCA E1p12 [35], consistent with the location of a nucleolar organizing region (NOR) [36]. Elevated argyrophilic nucleolar organizing region (AgNOR) scores, as defined by silver staining analysis, are common indicators of increased cellular proliferation, and have been associated with decreased overall survival for canine soft tissue sarcomas [37]. It is tempting to consider that this may reflect the inferior outcome that is typically associated with ISS. Statistical comparison of genomic profiles across the cohort identified two subchromosomal regions that showed significant differences in DNA copy number status in ISS *versus* non-ISS cases. While providing support for molecular discrimination, the limited resolution capabilities of the aneuploidy array confounded the ability to refine the boundaries of these regions and in turn to assess their gene content [35].

A higher-density second-generation cat CGH microarray platform has since been developed utilizing the 14x coverage cat genome sequence assembly, comprising ~110,000 oligonucleotide probes distributed at mean intervals of ~21kb. This provides a >300-fold increase in resolution over the aneuploidy array, bringing feline resources closely in line with those of other model systems for the first time. Evaluation of the same cohort of feline fibrosarcomas with this more comprehensive platform [38] identified homozygous deletions of the tumor-suppressor gene *PTEN* and recurrent amplifications of the *KIT* proto-oncogene. Elevated *KIT* protein expression has been reported as a persistent finding in both ISS and non-ISS tumors [39]; however it is not yet clear whether upregulation is a direct consequence of increased *KIT* gene dosage, nor whether this confers potential for utilizing targeted therapies directed towards this receptor tyrosine kinase. ISS and non-ISS tumors showed grossly comparable profiles of DNA copy number imbalance; however ISS cases exhibited elevated levels of global genomic disruption, consistent with their typically more aggressive behavior and poorer outcome. Among these lay a subset of discrete regions with promise as discriminatory markers [38]. With focus on the most clinicopathologically compelling genomic regions, in combination with assessment of transcriptional and translational status, there is great promise for a more detailed definition of the molecular pathogenesis of feline fibrosarcomas, and in turn, for refinement of diagnostic and therapeutic modalities for both subtypes. In a broader sense, genomic characterization of ISS also may aid elucidation of molecular mechanisms involved in the passage from chronic inflammation to neoplastic transformation, which continues to challenge human medicine.

## 6. Lymphoma

Lymphoma is the most common hematopoietic malignancy of cats and the third most common feline cancer overall, comprising approximately one third of all cancer diagnoses. Moreover, cats are considered to show the highest relative incidence of lymphoma among all species studied to date (reviewed in [40]), offering another promising angle for comprehending key facets of tumor biology. Historically, cytogenetic studies of feline lymphomas have focused heavily on established cell lines, primarily those developed from tumors associated with infection by feline leukemia virus, which often are nodal or mediastinal in origin. Common observations from these reports include hyperdiploidies resulting from low amplitude copy number increases of a variety of chromosomes, with no strongly recurrent features. These investigations have greatly expanded our understanding of the mechanisms by which clonal proviral integration into a host genome can initiate tumorigenesis and genomic instability through oncogenic activation [41,42]. With the diminished incidence of retrovirus-associated feline lymphoma since the 1980’s, as a result of increased testing, vaccination and global vigilance, the focus has since shifted toward subtypes that offer opportunities for a more comparative approach.

Extensive volumes of clinically predictive cytogenomic data are accumulating for nodal lymphomas in human patients, and more recently also in dogs [43,44]. In contrast, gastrointestinal (GI) lymphoma, which represents the most common extranodal form in people and comprises more than 50% of all feline lymphomas, remains vastly understudied at the molecular level [45,46,47,48]. Given the high incidence of the disease in cats there is an opportunity for feline studies to pave the way forward for mutual benefit. In cats, the overall survival time for low-grade small-cell GI-lymphomas is generally four-fold greater than for high-grade large-cell tumors, following standard of care chemotherapy [45,49]. The relative frequency of these histologic subtypes in different anatomical locations varies widely in prior reports [40,45,50,51,52], in part due to the logistical challenges of performing diagnostic evaluation of GI lesions by surgical biopsy [40,53]. This invites assessment of a genomic approach for comprehensive DNA-based subclassification. Initial observations from microarray-based CGH analysis have indicated that feline GI-lymphoma exhibits a relatively subtle degree of genomic copy number instability, with no evidence for recurrent high-level amplifications or homozygous deletions. The majority of aberrations are shared by fewer than 30% of cases, and include defects involving key cancer-associated genes, such as elevated copy number of the *MYC* proto-oncogene and deletion of *PTEN.* Efforts to identify candidates for molecular distinction between histologic and immunophenotypic subtypes are now underway [54]. 

## 7. Mammary Carcinoma

Mammary tumors represent the third most commonly diagnosed feline neoplasm, the vast majority of which (in contrast to dogs) are malignant, primarily simple carcinomas. Feline mammary carcinomas (FMCs) generally are highly aggressive, infiltrative and metastatic and show extensive heterogeneity in outcome that is not readily predicted at the time of diagnosis [55]. FMC shares extensive clinicopathologic, demographic and epidemiological similarity with human breast carcinoma (HBC), the most frequently diagnosed cancer in women [56]. While there is evidence for steroid hormone involvement in some FMCs, 80%–90% are estrogen- and progesterone-receptor negative [57,58,59]. FMC therefore offers a particularly powerful spontaneous model of the ~30% of “basal-like” late-stage HBCs that are highly aggressive and unresponsive to hormone-antagonist therapies, with a lack of targeted treatments and a poor prognosis. 

Diagnostic and prognostic descriptions for FMC frequently are limited in detail since there is little evidence to associate specific histomorphologic subtypes with outcome. In HBC histologic grade, typically defined using the Elston and Ellis scoring system, is one of the most robust prognostic factors [60]. Although inconclusive in dogs, it has been shown recently, using this same grading system, that invasive FMC also shows a significant incremental association between histologic grade and outcome [61]. In both human and feline patients, intermediate grade tumors show the lowest concordance in routine diagnosis and the broadest heterogeneity in outcome [60,61], and thus the greatest need for improved subclassification.

Genome-wide surveys of HBC have identified discrete genomic signatures that define clinically predictive subtypes, leading to effective treatment strategies based on molecular pathogenesis [62]. Assessment of evidence for corresponding molecular subtypes in FMC may confer potential to extrapolate these advances rapidly to feline patients. To date, the search for prognostic factors for FMC from a molecular perspective has been targeted to individual candidate genes known to be associated with HBC. Of particular note, approximately 25% of HBCs exhibit upregulation of the *ERBB2* gene encoding the receptor tyrosine kinase HER2/neu, of which >90% exhibit DNA copy number amplification of this region [63]. This aberration, historically considered to be an indicator of poor prognosis, also confers susceptibility to Trastuzamab (Herceptin^®^), a monoclonal antibody targeted against the HER2/neu receptor. Published estimates of HER2-positive FMCs by immunohistochemistry show tremendous variation between studies, largely due to the use of diverse patient cohorts and contrasting analytical techniques [64]. Consequently the true frequency of HER2-positive FMC, and the relationship between protein expression and gene copy number, is yet to be determined.

At the genome-wide level, early conventional chromosome banding studies of FMC identified highly disrupted karyotypes, including recurrent deletions of FCA B2 and gains of FCA E3 [65,66]. These long predated the “genomics era”, precluding detailed interpretation in terms of their significance in disease pathogenesis. Microarray-based molecular profiling of FMC is now yielding more substantial insight. Preliminary results with the second-generation CGH array have demonstrated moderate DNA copy number instability in FMC that is distributed broadly throughout the genome, with very few CNAs shared by more than 50% of cases surveyed [67]. Overall, higher-grade tumors exhibit an elevated incidence of genomic imbalance, suggesting that increased chromosome instability may itself represent an indicator of inferior outcome. Interestingly, copy number gain of *ERBB2* was >3-fold more common in high-grade *versus* intermediate-grade tumors. The incidence of *PTEN* deletion was also increased in high-grade tumors, correlating with an earlier study that showed inferior outcome in FMC cases with decreased PTEN protein expression [68]. Gain of *ERBB2* and deletion of *PTEN* are among the most recurrent genomic imbalances in HBC and are associated with concomitant disregulation of gene expression and anti-apoptotic activity, and in turn with inferior outcome [69]. Continued efforts should offer new insight into the degree to which the clinicopathologic similarities between HBC and FMC are recapitulated at the genomic level, highlighting disrupted pathways and potential therapeutic targets for FMC.

## 8. Conclusions

The domestic cat is becoming increasingly well placed to stimulate new advances in molecular medicine. The unique range and distribution of cancers diagnosed in our feline companions fills a series of critical niches that have confounded progress in human medicine. Included among this diverse spectrum are those with direct human counterparts that have remained understudied due to their rarity in people, while others offer a means to study discrete facets of tumor biology that are largely peculiar to the cat. We are now in a position to perform rapid and comprehensive interrogation of normal and aberrant feline genomes and to interpret our observations directly in terms of gene content and potential dysfunction. In turn we have the ability to extrapolate with relative ease between the genomes of different species in the search for conserved molecular features that suggest a common disease mechanism. Given the gross similarity in the structural organization and evolutionary history of the human and cat genomes, it is conceivable that an integrated cytogenomic approach may help to reveal as yet unrecognized mechanisms of tumor-associated chromosome instability.

The field of personalized medicine continues to make tremendous strides in the human field, and opportunities to integrate similar concepts into the veterinary arena are gradually becoming a reality. Cytogenomic techniques are continuing to evolve, with increases in resolution, sensitivity, flexibility, throughput and cost-effectiveness, coupled with the ability to combine related techniques into single platforms that allow synergistic acquisition of data from complementary approaches in a single experiment. In the human field, cancer cytogenomic discovery is gradually transitioning from chromosome- and microarray-based techniques toward the use of next generation sequencing approaches [17]. These strategies provide substantially increased resolution capabilities and have the advantage of permitting concomitant assessment of both structural and numerical aberrations in conjunction with detection of sequence mutations. At the present time, however, their integration into the veterinary arena is constrained by high costs and intensive computational needs, and limited access to dedicated reagents and analytical tools for non-human species. Ten years ago, the same limitations applied to the use of microarray-based technologies such as those described here. Given that the field of veterinary molecular oncology is now well established, and its comparative and translational value is continually being proven, one must hope that the delay in widespread introduction of next generation sequencing strategies for use in companion animal medicine will be less profound.
